# All-Oral Antibiotic Treatment for Buruli Ulcer: A Report of Four Patients

**DOI:** 10.1371/journal.pntd.0000770

**Published:** 2010-11-30

**Authors:** Claire L. Gordon, John A. Buntine, John A. Hayman, Caroline J. Lavender, Janet A. M. Fyfe, Patrick Hosking, Mike Starr, Paul D. R. Johnson

**Affiliations:** 1 Department of Infectious Diseases, Austin Health, Melbourne, Australia; 2 Department of Surgery, Box Hill Hospital, Melbourne, Australia; 3 Department of Surgery, Monash University, Melbourne, Australia; 4 Department of Anatomy and Developmental Biology, Monash University, Melbourne, Australia; 5 WHO Collaborating Centre for Mycobacterium ulcerans (Western Pacific Region) and Victorian Infectious Diseases Reference Laboratory, Melbourne, Australia; 6 Department of Anatomical Pathology, Box Hill Hospital, Melbourne, Australia; 7 Department of Infectious Diseases, Royal Children's Hospital, Melbourne, Australia; Emory University, United States of America

## The Cases

**Figure 1:**
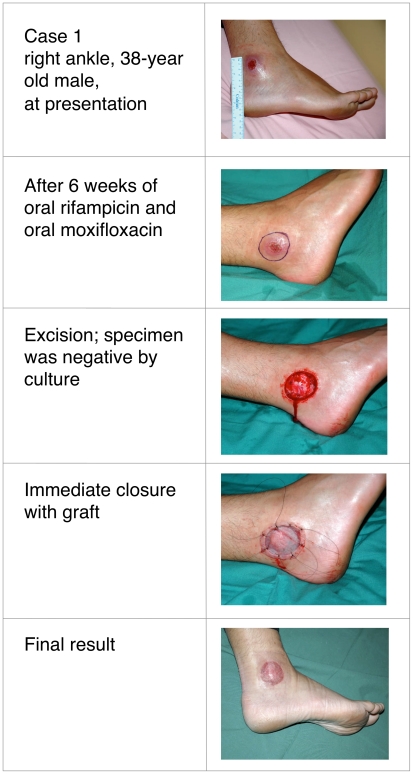
Thirty-eight-year-old male with culture confirmed Buruli ulcer before, during, and after treatment.

Buruli ulcer (BU) was treated primarily with wide surgical excision until recent studies confirmed the efficacy of oral rifampicin combined with intramuscular streptomycin. Whether all-oral antibiotic regimens will be equally effective is unknown. This report describes four patients with *Mycobacterium ulcerans* infection, all of whom received rifampicin-based oral antibiotic therapy followed by surgical resection (three patients) or oral antibiotics alone (one patient). Following oral antibiotics for between 4 and 8 weeks, viable *M. ulcerans* was not detectable by culture in three of the patients, or by histology in a fourth patient from whom no specimen for culture was obtained. All cases spent time in a BU-endemic area in coastal Victoria, Australia. Baseline characteristics, diagnosis, treatment received, and histopathology of resected specimens are detailed in Table 1. Clinical photographs are shown in Figures 1–4. All patients gave informed consent for publication.

**Figure 2 pntd-0000770-g002:**
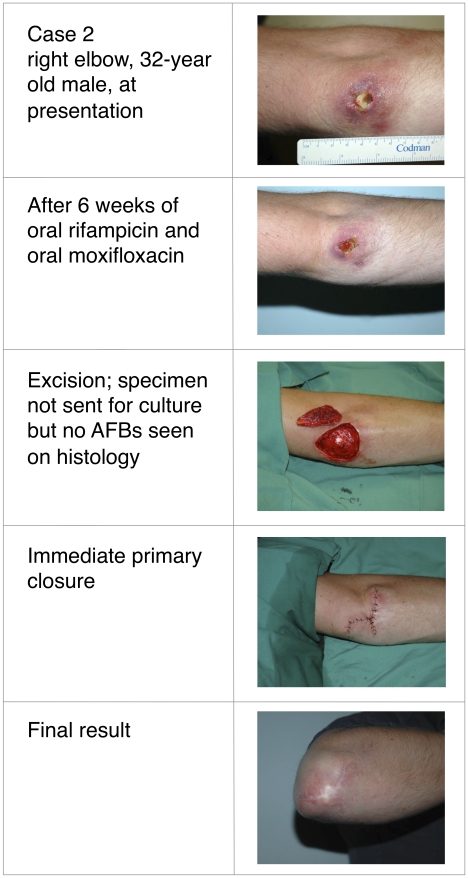
Thirty-two-year-old male with culture confirmed Buruli ulcer before, during, and after treatment.

**Figure 3 pntd-0000770-g003:**
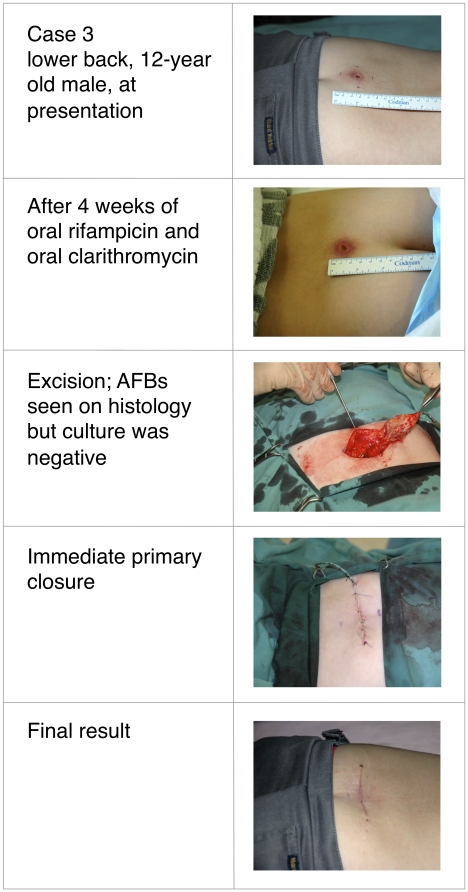
Twelve-year-old male with culture confirmed Buruli ulcer before, during, and after treatment.

**Figure 4:**
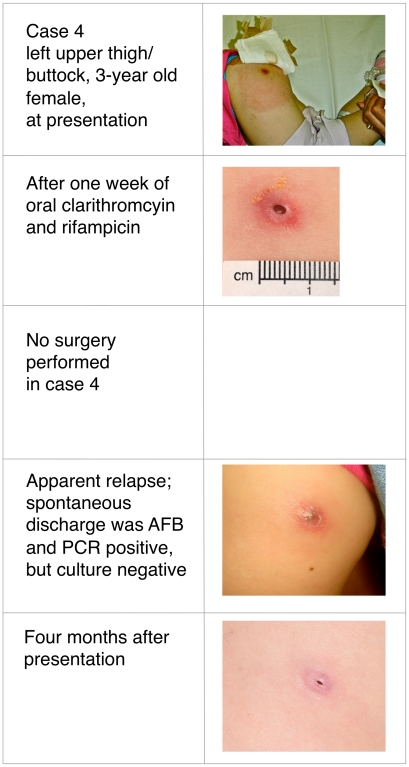
Three-year-old female with culture confirmed Buruli ulcer before, during, and after treatment.

**Table 1:** Baseline Characteristics, Diagnosis, Treatment Received, and Histopathology and Microbiology of Resected Specimens.

	Case 1	Case 2	Case 3	Case 4
*Age*	38-year-old male	32-year-old male	12-year-old male	3-year-old female
*Location of lesion*	Right lateral malleolus	Right elbow	Lower back	Left thigh/buttock
*Clinical form*, *size*, *and WHO category of lesion* [17]	Ulcer, 2cm diameter, category 1	Ulcer, 2cm diameter, srcategory 1	Pre-ulcerative form, 4cm diameter of induration, category 1	Ulcer, 2cm diameter, category 1
*Region exposed*	Bellarine Peninsula	Bellarine Peninsula	Bellarine Peninsula	Bellarine Peninsula
*Specimen collected for diagnosis*	Dry swab	Dry swab	Saline-moistened swab	Dry swab
*Basis of diagnosis*	PCR and culture	PCR and culture	PCR and culture	PCR and culture
*Date of laboratory diagnosis*	November 2006	October 2008	November 2008	November 2009
*Principal drug*	Rifampicin 600 mg daily	Rifampicin 600 mg daily	Rifampicin 450 mg daily reduced to 600 mg 3× week after day 7	Rifampicin 10mg/kg daily
*Secondary drug*	Moxifloxacin 400 mg daily	Moxifloxacin 400 mg daily	Clarithromycin 250 mg twice daily reduced to 250 mg twice daily, alternate days after day 7	Clarithromycin 15mg/kg daily in divided doses
*Duration of oral drug therapy prior to excision*	6 weeks	6 weeks	4 weeks	8 weeks (lesion not excised)
*Outcome (follow-up period)*	No recurrence (36 months)	No recurrence (13 months)	No recurrence (12 months)	Improved to match head sized palpable nodule
*Histology/microbiology summary of excised specimen (cases 1–3; no excision case 4)*	No AFB, culture negative, chronic granulomatous inflammation without necrosis	No AFB, chronic necrotizing granulomatous inflammation, culture not performed	AFB seen, culture negative, necrosis to edges of excision	Spontaneous discharge 4 weeks after ceasing antibiotics, AFB seen, PCR positive, culture negative
*Comment*			Doses and duration reduced due to drug intolerance	Apparent relapse due to a culture negative “paradoxical” reaction [22]

In all patients, the diagnosis of *M. ulcerans* was confirmed by positive polymerase chain reaction (PCR) and isolation of *M. ulcerans* by culture from swabs obtained prior to treatment. Three patients had ulcerative lesions (Table 1: cases 1, 2, and 4; Figures 1, 2, and 4) and one had a pre-ulcerative lesion (Table 1: case 3 and Figure 3) from which a saline-moistened swab of the lesion yielded a positive PCR and culture. For the two adults (Table 1: cases 1 and 2), rifampicin was combined with moxifloxacin for 6 weeks prior to resection. The two children (Table 1: cases 3 and 4) received rifampicin combined with clarithromycin for either 4 weeks prior to resection (Table 1: case 3) or 8 weeks without resection (Table 1: case 4). In cases 1 and 3, resection specimens were culture-negative, and culture was not performed in case 2, although histology showed resolving inflammation and no acid-fast bacilli (AFB) by Ziehl-Neelsen staining. In case 3, a Ziehl-Neelsen stained section showed persistent AFB but culture was negative. He had received a reduced dose of rifampicin due to gastrointestinal and neurological side effects and underwent earlier excision than planned at 4 weeks. Inflammation of surrounding skin and the size of the lesion reduced during antibiotic therapy in all four patients (Figures 1–4). PCR was not performed on surgical excision specimens (cases 1–3). Excision and primary closure, rather than grafting, was achieved in case 2 and 3, which had not been considered possible initially. Antibiotic treatment was continued after surgery in all patients. Total treatment duration was 7 weeks for case 3 and 12 weeks for cases 1 and 2. Case 4 was a 3-year-old girl who was treated with oral combination antibiotics without surgery. After 8 weeks of rifampicin and clarithromycin syrup, the ulcer had reduced to a very small palpable nodule. However, 4 weeks after ceasing antibiotic therapy the lesion became inflamed and discharged pus. Acid-fast bacilli were seen and PCR for *M. ulcerans* was positive. However, subsequent culture was negative at 3-months, suggesting an immune-mediated “paradoxical reaction” driven by residual but dead mycobacterial cells, rather than a true failure of oral antibiotic therapy. Following spontaneous discharge only a small blind ending sinus remained.

## Discussion

BU is a slowly progressive and destructive soft tissue infection, with the potential for severe scarring and disability [1], [2]. The main burden of disease occurs in sub-Saharan Africa [1], although in Australia, there are also active foci in coastal Victoria, the Daintree region in the far north, and near Rockhampton, Queensland [3]–[6].

Until recently, the practice of wide surgical excision followed by grafting has been the mainstay of treatment [2]. High relapse rates [7], prohibitive cost, and limited access to surgery in endemic areas in Africa led to a renewal of interest in antibiotic therapy, which had not appeared effective when first studied in field trials [8]–[10]. Based on promising experiments in the mouse footpad model [11]–[15], a small pilot study established that the combination of oral rifampicin and intramuscular (IM) streptomycin for 8 weeks was able to sterilize early BU lesions in humans. In this small study of 21 pre-ulcerative patients in Ghana, even 4 weeks of rifampicin and streptomycin led to culture negativity when lesions were surgically excised after antibiotic treatment of varying durations [16]. Based on this result, WHO introduced and promoted a new protocol of initial therapy with 8 weeks of daily oral rifampicin and IM streptomycin for all patients with BU, although it was expected that many would still require surgery [17]. Subsequently, Chauty et al. reported a case series of 224 patients with pre-ulcerative and ulcerative BU who were treated with this regimen [18]. Of the 215 patients whose lesions healed, 47% were treated only with antibiotics and did not require surgery. Although there were no microbiological studies, recurrence of *M. ulcerans* infection occurred in only two patients treated with antibiotics alone. In a recent randomized trial of 151 patients, the majority of whom also did not have surgery, Nienhuis et al. demonstrated that oral rifampicin plus IM streptomycin for 4 weeks then oral rifampicin plus oral clarithromycin for 4 weeks was as effective as 8 weeks of oral rifampicin plus IM streptomycin [19], indicating that a shorter duration of IM streptomycin is also effective.

In Australia, surgery is widely accessible and remains the main treatment modality for BU, although often in combination with oral antibiotics. As a result, the efficacy of surgery alone compared with oral antibiotics alone is difficult to establish, although relapses may be less when both modalities are used [6], [20], [21]. Australian consensus guidelines [6], now 4 years old, recommend surgery alone for small lesions or surgery combined with antibiotic therapy for more extensive disease. These guidelines include the use of IV amikacin for severe disease, but in practice amikacin is rarely used due to concerns about ototoxicity. Other oral antibiotics that appear to be active against *M. ulcerans* in mice include moxifloxacin and clarithromycin [11]. Clarithromycin is preferred in children due to its established safety record. There are unpublished accounts of successful treatment of BU with oral rifampicin alone (W. Meyers, personal communication), and the first published report of successful use of oral antibiotics was of a North Queensland farmer with acute, oedematous *M. ulcerans* disease who received oral rifampicin, clarithromycin, and ethambutol for 12 weeks immediately following extensive but incomplete surgical excision [20]. The surgical resection margin showed AFB, but further biopsies taken after 3 weeks of antibiotics due to concern about relapse were smear and culture negative. In retrospect, this apparent clinical deterioration may have been a “paradoxical reaction” [22] and the case demonstrated the principle that oral antibiotics are able to prevent relapse after incomplete surgical excision, even in a severe form of BU.

In the four patients we have described here, combination oral antibiotic therapy prior to excision led to the inability to recover *M. ulcerans* by culture in the three cases from whom a second specimen was submitted for culture, confirming that oral combinations of antibiotics are capable of sterilizing lesions in humans, as Etuafal et al. demonstrated for rifampicin plus IM streptomcyin. Two of the three patients we described received oral antibiotics for a total of 12 weeks. However, negative culture results at excision (4 weeks for case 3, 6 weeks for case 1) suggest that shorter periods may be effective as suggested for the combination of rifampicin and streptomycin [16], [17]. Dossou et al. also reported clinical improvement after 8 weeks of oral rifampicin and clarithromycin [23] in a pregnant patient. However, in all patients we have described, the clinical appearance of the lesions only improved slowly over several weeks. As experience with antibiotics increases it has become apparent that healing is slow but continues long after the treatment course is completed [18], [19]. Although this is a small clinical case series of Category I BU, oral rifampicin in combination with clarithromycin or moxifloxacin shows promise and should be further investigated.

**Key Learning Points**

- Treatment of patients with limited BU prior to surgery using rifampicin-based oral antibiotics resulted in culture-negative resection specimens.
- Clinical healing is slow despite the microbiological activity of oral antibiotics.
- Apparent relapses that occur during or after treatment may be due to immunologically driven paradoxical reactions rather than primary treatment failure.
- Rifampicin-based oral antibiotic therapy for the treatment of *M. ulcerans* infection followed by delayed surgery appears to simplify management by allowing excision and closure in one step without relapse.
